# 527 Combination Treatment for Hypertrophic Burn Scars Utilizing Fractional Ablative and Continuous Wave CO2 Laser

**DOI:** 10.1093/jbcr/irae036.162

**Published:** 2024-04-17

**Authors:** Punhae Lee

**Affiliations:** Hangangsoo plastic surgery, Seoul, Seoul-t'ukpyolsi

## Abstract

**Introduction:**

Hypertrophic scars remain a devastating consequence of severe burn wounds, producing anatomic deformities, as well as functional impairment. Fractional ablative CO2 laser treatment has shown scar - reducing effects. In this study, we compared the outcomes of combining two different CO 2 lasers or fractional ablative CO2 laser alone in treating hypertrophic burn scars.

**Methods:**

Forty patients with hypertrophic burn scars were included in one of following two groups: fractional ablative CO2 laser combined with continuous wave CO2 laser (group 1) or fractional ablative CO2 laser alone (group 2). Three plastic surgeons rated the hypertrophic scars according to observer-rated Vancouver Scar Scale (VSS) before and after treatment (at least 6 months after the last laser) and by patient-completed questionnaires after treatment (at least 6 months after the last laser).

**Results:**

Forty patients completed the laser treatment. Group 1 showed significant improvement in vascularity, pliability, and height than group 2 (p < 0.05) but not in pigmentation. Time-dependent analysis of total VSS scores suggested that group 1 experienced more improvement during a shorter treatment period than group 2 (p < 0.05). For patient-reported outcomes, group 1 showed significant improvement than group 2 in scar appearance, scar thickness, pain, and pruritus (p < 0.05).

**Conclusions:**

Observer-reported VSS outcomes showed that combining laser treatment resulted in more improvement of vascularity, pliability, and height indices for hypertrophic burn scars. Patient-completed outcomes showed that combining laser therapy was considered more helpful for improving scar appearance, scar thickness, pain, and pruritus than fractional ablative laser alone. Furthermore, combination treatment appeared to produce more improvement in a shorter period of time.

**Applicability of Research to Practice:**

The two lasers are widely used in scar treatment in South Korea, so yes this research can be applied to medical practice.

